# Functional Food for Elderly High in Antioxidant and Chicken Eggshell Calcium to Reduce the Risk of Osteoporosis—A Narrative Review

**DOI:** 10.3390/foods10030656

**Published:** 2021-03-19

**Authors:** Marcellus Arnold, Yolanda Victoria Rajagukguk, Anna Gramza-Michałowska

**Affiliations:** Department of Gastronomy Science and Functional Foods, Faculty of Food Science and Nutrition, Poznań University of Life Sciences, Wojska Polskiego 31, 60624 Poznań, Poland; marcellusarnold95@gmail.com (M.A.); yola.victoria.raja@gmail.com (Y.V.R.)

**Keywords:** elderly, antioxidant, chicken eggshell, functional food, osteoporosis, bread, food waste

## Abstract

The elderly population is increasing globally and is predicted to reach 1.5 billion in 2050. The quality of life of the elderly must be concerned, for example, with developing functional food for the elderly. In this article, the development of functional food to reduce the risk of osteoporosis in the elderly is reviewed. Oxidative stress is one of the factors which accelerates osteoporosis. Various antioxidants, including vitamin C, vitamin E, polyphenols, or lycopene, have been proven by former studies to have antioxidant activity, therefore, could reduce the risk of osteoporosis. Additionally, the application of eggshell powder in various food products has been reported to improve calcium intake, and its usage is environmentally sustainable as this could contribute to reducing food waste. The application of both antioxidants and calcium could be a good combination, but the amount of some antioxidants must be concerned so it would not interfere with the bioavailability of calcium. Therefore, this review aims to explore the functional food for the elderly to reduce the risk of osteoporosis, particularly with antioxidants and calcium from chicken eggshells. The eating preference and dietary pattern of the elderly are also considered to determine the suitable form of functional food for the elderly. The results presented in the study may be the basis for the development of new calcium-enriched food products for the elderly.

## 1. Introduction

The number of the elderly, or people aged 65 years or over, is increasing year-by-year. Globally, a total of 703 million elderly was reported in 2019, with the highest number of the elderly was found in Eastern and South-Eastern Asia (260 million), followed by Europe and Northern America (over 200 million). In 2050, the number of elderly in the world will double to 1.5 billion. The shared population of the elderly in 1990 and 2019 was 6% and 9%, respectively, and it is projected to rise further to 16% in 2050. This means that in 2050, one in six people will be 65 years old or over [1]. In 2019, the population of the elderly aged 65 years and over in the European Union-27 was more than one-fifth of the population (20.3%), an increase of 2.9% compared with the elderly population in 2009 (17.4%) [2].

Due to the increasing number of the elderly, some efforts to support the life quality of the elderly must be concerned, including osteoporosis, which is a public health problem. Currently, it has been estimated that osteoporosis affects more than 200 million people in the world [3]. In worldwide, one in three women with age over 50 years and one in five men will experience osteoporotic fractures in their lifetime [4]. Approximately one-tenth of women aged 60, one-fifth of women aged 70, two-fifths of women aged 80, and two-thirds of women aged 90 suffered osteoporosis [5]. In the United States and Europe, around 30% of postmenopausal women suffer osteoporosis, with more fragility fractures suffered by 40% of them [6].

To reduce the risk of various health problems in the elderly, a review based on recent research reported that the elderly need functional foods containing dietary fiber, phytoestrogens, omega-3 polyunsaturated fatty acid, polyphenols, carotenoids, lutein, zeaxanthin, prebiotics, probiotics, synbiotics, plant sterols, and stanols [7]. Antioxidants were reported to reduce oxidative stress, which can be beneficial for reducing the risk of osteoporosis [8].

In addition to health problems, solving environmental problems, including food waste and loss, is also important. Food waste and food loss currently have been the challenging issue in the world in the prospect of feeding a population of nine billion by 2050. Food and Agriculture Organization of the United Nations [9] reported that reducing food loss and waste could be an important way to lower production costs, improve food security and nutrition, and contribute towards environmental sustainability by easing the pressing on natural resources and decreasing greenhouse gas emission. Food loss and waste are defined as the edible parts from plants and animals, which are produced or harvested across the food supply chain for human consumption, but that is not ultimately consumed by people [10]. Food loss refers to the decrease of food quantity or quality, which makes it unfit for human consumption, while food waste refers to the good quality food and fit for human consumption that does not get consumed because it is discarded either before or after it spoils [10,11]. Food waste also includes edible material, which is intentionally fed to animals or is a byproduct of food processing diverted away from human food [12]. In sub-Saharan Africa and South/Southeast Asia, the food waste at the consumption stage ranged from 6–11 kg/person/year, while in North America and Europe ranged from 95–115 kg/person/year [13]. Among the European countries, a large amount of food waste per person per year were found in Netherlands (579 kg), Belgium (399 kg), and Cyprus (334 kg), while the smallest was found in Greece (44 kg), Malta (62 kg), and Czech Republic (71 kg) [14]. In Poland, approximately 235 kg of food is wasted per person annually [15]. In Arab countries, the food loss and waste reached the amount of 210 kg/person/year [16].

Food wastes such as chicken eggshells [17], duck eggshells [18], quail eggshells [18], seashells [19], oyster shells [20], and fish bones [21] have been reported to have high contents of calcium, which is important to prevent osteoporosis. The potential uses of eggshell are classified into raw material for new products manufacture and operating supply [22]. In raw material, eggshell can be utilized as a food additive, soil amendment, purified calcium carbonate (CaCO_3_), cosmetics, and biomaterial composite. While in operating supply, eggshells can be used as catalysts and sorbents.

Food is one of the important factors, which influences a healthy lifestyle, which must be concerned by the elderly [23]. The most advantageous method of supplementing calcium deficiency in the body is its supply of food. However, in many cases, it is not enough, and therefore, other alternative sources of easily digestible forms of calcium are constantly searched for. It is worth noting, however, that chicken eggshells are a very good natural source of calcium, with high potential for use in food. Therefore, this review aims to explore the functional food for the elderly to reduce the risk of osteoporosis, particularly with antioxidants and calcium from chicken eggshells.

## 2. Bone and Osteoporosis

Bone is the main calcified tissue of vertebrates, which serves multiple functions, such as mechanical support, protection, and storage [24,25]. Bone consists of 10% cells, 60% mineral crystals (crystalline hydroxyapatite), and 30% organic matrix [26]. Osteoblast and osteoclast are the main cells, which are responsible for bone remodeling [27]. The osteoblast is a bone-forming cell, which provides signals for osteoclast formation, while the osteoclast is specialized multinucleated giant cells, which resorbs bone [25]. In bone remodeling, osteoclasts remove the old or damaged bone, and osteoblasts form the new bone over several weeks [24]. Some factors, which influence the activities of bone cells are nutritional and cellular factors, including the supply of oxygen, nutrients, endocrines, cytokines, growth factors, and free radicals [28]. If there is an imbalance between the activities of osteoblast and osteoclast, it would ultimately lead to osteoporosis [8].

Osteoporosis is a health problem characterized by low bone mineral density, deterioration of bone microarchitecture, decreased bone mass, increased bone fragility, and it is considered an age-related disorder [24,29]. Osteoporosis could increase the risk of fragility fracture and could give a negative effect on the quality of life in populations. The risk factors of osteoporosis can be divided into unmodifiable and modifiable factors, where oxidative stress-related factors (in which the low antioxidant status) are included in modifiable factors [30]. The important factor, which leads to osteoporosis is also the lack of calcium intake, which leads to a suboptimal bone mass peak and low bone mineralization [6]. Results of Cao and Rana et al. demonstrated that also childhood obesity is associated with severe co-morbidities, including bone loss [31].

The prevalence of osteoporosis was reported by prior studies. The elderly are at a greater risk of osteoporosis despite the preventive treatments are available [32]. Osteoporosis is more common in women than men, and its prevalence increases significantly after menopause [6]. Postmenopausal osteoporosis, which is associated with deficiency of estrogen, an important hormone influencing bone mineral density, leads to bone loss through increased osteoclastic function [28]. In recent years, new evidence has emerged of the link between the immune system and bone diseases, such as rheumatoid arthritis and osteoporosis [33,34] demonstrated that ovariectomy induces the T-cell costimulatory immunomodulatory cytokine LIGHT (lymphotoxin-like inducible protein), which further stimulates osteoblastogenesis and osteoclastogenesis by modulation of osteoclastogenic cytokine expression. LIGHT was found to mediate ovariectomy-induced bone loss, suggesting a positive effect of LIGHT antagonism in patients with postmenopausal osteoporosis.

The prevalence of osteoporosis in women aged 50 years and above was 9% in the United Kingdom, 15% in France and Germany, 16% in the USA, and 38% in Japan. For males aged 50 years and above was 1% in the United Kingdom, 4% in Japan, 3% in Canada, and 8% in France, Germany, Italy, and Spain [35]. Elderly males with anemia and prior fracture are associated with a higher risk of osteoporosis [36]. Around 80% to 90% of Polish patients suffering from osteoporosis, including osteoporotic fractures, do not receive sufficient pharmacotherapy [37].

### 2.1. Role of Oxidative Stress in Osteoporosis

Oxidative stress is one of the risk factors for osteoporosis. The imbalance between free radical production and antioxidant capacity may cause oxidative stress, and this oxidative stress leads to the pathogenesis of various chronic diseases [28]. Free radical is an atom or molecule with a single unpaired electron, for example, superoxide anion (O_2_^•−^), hydroxyl radical (^•^OH), lipoperoxide radical (LOO^•^) [38]. Reactive oxygen species (ROS) or reactive nitrogen species (RNS), including peroxynitrite (ONOO^−^), nitric monoxide (NO^•^) and nitrogen dioxide (NO_2_^•^), are the radicals produced in vivo [38]. Oxidative stress may induce osteoporosis through the increased expression of cytokines in the bone [28]. The resorption of bone by oxidative stress undergoes through the activation of the nuclear factor-κB protein, which is a crucial mediator of tumor necrosis factor-α (TNF-α) and osteoclastogenetic activity [39].

During normal metabolism, ROS are produced by the activation of various enzymes, such as the nicotinamide adenine dinucleotide phosphate oxidase or NADPH oxidase (membrane enzyme), superoxide dismutase (cytoplasmic enzyme) and various mitochondrial oxidases [8]. ROS, for example, superoxide and hydrogen peroxide, are the regulatory factors in osteoclastic resorption bone activity, and the generated superoxide from osteoclast contributes to bone degradation [28]. Production of ROS in osteoclasts can be increased more in some conditions, such as deficiency of vitamin D, fractures, increasing age, which then leads to osteoporosis [28]. ROS have an extremely short half-life, so they are difficult to be measured. The only way to measure ROS is through the damage which they cause to proteins, lipids and DNA, which is revealed as chronic diseases, including osteoporosis [27,40].

### 2.2. Effects of Antioxidants in Osteoporosis

Antioxidants can be protective against oxidative stress and are the potential to reduce the risk of osteoporosis. Antioxidants contribute to activating the differentiation of osteoblast, mineralization and osteoclast activity reduction, either directly or by counteracting the action of oxidants [8]. Endogenous enzymes in our body (i.e., antioxidant enzymes glutathione peroxidase, catalase, superoxide dismutase, and metal-chelating proteins) and exogenous antioxidants (i.e., polyphenols, carotenoids, vitamin C, and vitamin E) from dietary sources present in fruits and vegetables were studied to lower the risk of oxidative damage [27]. Wilson et al. [41] reviewed the effects of antioxidants and the benefits of functional foods on the health of the elderly. They studied that antioxidants have important roles in treating chronic diseases related to the elderly, for example, Alzheimer’s disease, diabetes, cardiovascular disease, cancer, and digestive system problems. However, the effects of antioxidants on osteoporosis in the elderly were not reviewed in this article. The effect of lycopene and other antioxidants on the prevention and treatment of osteoporosis, especially in postmenopausal women, was also studied [27].

#### 2.2.1. Vitamin C

Vitamin C, as a primary antioxidant, is able to remove ROS and RNS, then decreases oxidative stress, which is related to osteoporosis [3,39]. Low ascorbate intake can reduce bone mass, a greater rate of bone loss, and can increase fractures [42]. The supplementation of vitamin C may provide changes regarding improvement in oxidative stress and bone mineral density [43]. Kim and Lee [3] reported that a higher intake of vitamin C could lower the risk of osteoporosis in Korean adults aged over 50 years with low physical activity, but no significant effect was found in those with high physical activity. Furthermore, higher dietary vitamin C intake was connected with a lower risk of hip fracture and osteoporosis, and also related to higher bone mineral density, particularly at the femoral neck and lumbar spine [44].

Earlier studies have shown the role of vitamin C in reducing the risk of osteoporosis by enhancing the formation of collagen [28,42,45], increasing calcium absorption [3,46], giving a possible effect on bone formation [28], and limiting the excessive free radicals formation [39,47]. Greater amounts of collagens were obtained at vitamin C concentrations of 200 μg/mL compared to 25 and 100 μg/mL [48]. Vitamin C is an essential activator of enzymes required for the hydroxylation of proline and lysine residues within collagen fiber, and this hydroxylation reaction allows covalent bond synthesis between the amino acid residues, resulting in strength improvement of overall collagen [39,45].

Vitamin C may not only exhibit antioxidant properties but may also exhibit prooxidant traits when consumed at higher concentrations, as the prior study found that 500 mg/day of vitamin C supplementation in men and women (aged between 17 and 49) for 6 weeks could promote oxidative DNA damage [49], and this may also be relevant to osteoporosis [45]. Furthermore, vitamin C at first was reported to act as an oxidant during osteoclastogenesis, and the oxidative stress accelerated osteoclast formation, but it caused osteoclast death at a later stage [50,51]. The main sources of vitamin C in the human diet are citrus fruits, pepper and kiwifruit, as well as cabbage, broccoli, kale or tomatoes [52]. Therefore, the food market is also offering numerous products fortified with vitamin C.

#### 2.2.2. Vitamin E

Vitamin E is an antioxidant with two major subgroups: tocopherols and tocotrienols. Each of these has four distinct analogs (alpha, beta, gamma, and delta) [53]. Both vitamin C and the antioxidant vitamin E show osteoprotective effects [28,47]. Vitamin E is a free radical scavenger, and deficiency of vitamin E impairs calcium transport via the intestine and reduces bone density [28]. Vitamin E and C limit the excessive free radicals formation and thereby controlling Malondialdehyde (MDA) levels, which indicate the lipid peroxidation in the body [47]. The increased activity of osteoclasts leads to the increased free radical formation and hence lipid peroxidation.

The α-tocopherol is one of the homologs of vitamin E. The α-tocopherol has a high interest as it is a potent lipoperoxyl radical scavenger. However, based on current studies, vitamin E homologs potentially impact differently on bone indices; although studies demonstrated that vitamin E is beneficial for bone via anti-inflammatory properties, this relationship has not been shown in humans [54].

Some studies reported both positive and negative effects of vitamin E in the elderly. Shi et al. [55] examined the relationship between vitamin E (in both serum and diet) and bone mineral density among middle-aged and elderly Chinese adults (aged 40–75 years) in Guangzhou. The results concluded that greater consumption and higher serum levels of vitamin E are associated with greater bone mineral density in Chinese women, but not in Chinese men. However, another study in elderly men living in Uppsala, Sweden, demonstrated that vitamin E (α-tocopherol) is able to maintain their bone mineral density [56]. Furthermore, the negative association between serum α-tocopherol concentration and femoral neck bone mineral density in the US elderly population (men and women, aged 50 years and older) was reported, suggesting a harmful effect of α-tocopherol on bone health [53].

#### 2.2.3. Polyphenols

Polyphenols, as well as lycopene (carotenoid), have beneficial effects to prevent the development of chronic disease caused by oxidative stress, including osteoporosis, diabetes, cancer, cardiovascular disease, and asthma [27]. Consuming fruits and vegetables can increase the intake of these antioxidants.

Polyphenols can be naturally found in plants (fruits, vegetables, grains, spices and herbs), and they are water-soluble [27,57]. Polyphenols can be divided into three main subgroups: phenolic acids, flavonoids and non-flavonoids [57]. Phenolic acids are hydroxyl derivatives of aromatic carboxylic acids with a single phenolic ring and further can be divided into two main types, benzoic acids and cinnamic acids. Phenolic acids contribute around 30% of the free and bound forms of dietary phenolics in plants. Flavonoids contain two phenolic rings, which are linked by a three-carbon bridge that is usually an oxygenated heterocycle. Polyphenols are good electron or hydrogen atom donors, thus can neutralize free radicals and other ROS due to their aromatic features and highly conjugated system with multiple hydroxyl groups [57]. The polyphenols intake was proven to give beneficial effects on chronic diseases, including osteoporosis, cancer, cardiometabolic risk, etc. [58].

Studies about polyphenols and their role in reducing the risk of osteoporosis were reported. A meta-analysis based on 17 journal articles using the keywords “tea and osteoporosis” was reported [59], concluding that consuming tea that contains polyphenols could reduce the risk of osteoporosis in all examined subgroups. Dietary intake of polyphenols in red wine extract resulted in a remarkable effect on bone strength improvement and prevention of osteopenia in estrogen-deficient ovariectomized rats [60]. Shen et al. [61] reviewed the effect of polyphenols-rich fruits (dried plum, citrus fruits, berries, and grapes) in bone protection.

The antioxidative effects of polyphenols in berries to reduce age-related bone loss was reported by Hubert et al. [62], indicating that berries are the possible cheap alternatives to reduce the risk of osteoporosis. Among the berries, the antioxidative effects of blueberry [63,64,65], chokeberry [66,67,68,69], cranberry [69,70,71], and goji berry [72,73] in reducing the risk of osteoporosis were reported. In recent years, new evidence has emerged of the health-promoting effects of sweat cherries on bone impairment associated with childhood obesity [74,75] demonstrated that obese children and adolescents bone impairment is sustained by spontaneous osteoclastogenesis, which can be limited in vitro by polyphenols presence. Research conducted on cultured peripheral blood mononuclear cells (PBMCs) from obese children showed that sweet cherry extract reduced the spontaneous formation of multinucleated osteoclasts in examined cells. It was reported that proper extraction of fruits, e.g., sweet cherry, and its application as a nutraceutical food offers high potential as preventive both in healthy children and therapeutic in obese.

Tea, in general, is a popular beverage in the world as it provides an attractive aroma, typical taste, health promotion and pharmaceutical potential [76]. Tea contains catechins that have four main monomers, such as (-)-epigallocatechin-3-gallate (EGCG), (-)-epigallocatechin (EGC), (-)-epicatechin-3-gallate (ECG), and (-)-epicatechin (EC) [55]. In a case–control study, a significant risk reduction of osteoporotic hip/femur fractures in middle-aged and elderly men who have tea-drinking habits was reported [77]. Green tea is also rich in polyphenols, which can increase antioxidant activity [78]. A cross-sectional study reported that green tea drinking habit was associated with increased bone mineral density in elderly Japanese women [79]. It was suggested that EGCG, one of the major flavonoids in green tea, induces the apoptosis of osteoclasts, then inhibits bone resorption, and may lead to increased bone mineral density [79]. In middle-aged female rats study, the protective effect of polyphenols of green tea on bone loss by decreasing oxidative stress damage was also reported [80].

However, too high a concentration of tea during tea-drinking habits weakens the protection from osteoporosis due to the high content of caffeine (1–4% of the dry weight of tea). Besides increasing urinary calcium excretion and reducing the intestinal absorption of calcium, the caffeine also acts as an agonist of adenylate cyclase (route C) through the phosphodiesterase’s activity inhibition, which affects the bone [77]. The caffeine intake of more than 300 mg/day accelerates spine bone loss in elderly postmenopausal women [81]. Nevertheless, as long as the individuals ingest the daily allowances of calcium based on current recommendation, no harmful effect of caffeine-containing beverages on bone status was reported [82]. Furthermore, tannic acid also reduces calcium absorption [83]. Therefore, the amount of polyphenols in the food products must be concerned, so it could give a positive and optimum impact on osteoporosis.

#### 2.2.4. Lycopene

Lycopene is one of the carotenoids, which is an acyclic isomer of β-carotene and cannot form vitamin A due to lack of β-ionone ring structure [84]. It is a highly unsaturated hydrocarbon containing 13 double bonds, including 11 conjugated and 2 unconjugated double bonds. This conjugated double bond chain determines their biological functions, which include a scavenger activity on free radicals [85]. Lycopene is considered as one of the most potent carotenoids due to its singlet-oxygen-quenching ability twice higher than β-carotene and 10 times higher than α-tocopherol [84]. Not only tomatoes but also watermelons, papayas, pink grapefruits and guavas contain lycopene [86].

Several studies related to lycopene’s influences in osteoblast and osteoclast were reported. Lycopene could inhibit the formation of multinucleated osteoclast cells and the formation of ROS-secreting osteoclast [87]. Ishimi et al. [88] also studied the effect of carotenoids, including lycopene, on osteoclast formation and bone resorption in murine osteoclasts formed in co-culture with calvarial osteoblasts. In a rat study, it was reported that lycopene treatment in ovariectomized rats primarily suppressed bone turnover to restore bone strength and microarchitecture [89]. A study found that intake of 10 mg/kg of lycopene for 60 days decreased the bone loss in femur epiphysis in ovariectomized rats by maintaining trabecular bone similar to controls [90]. After 17 years of follow-up, elderly men and women aged 75 years with higher lycopene intake were reported to have a lower risk of hip fracture (*p* = 0.01) and non-vertebral fracture (*p* = 0.02) [91]. In a cross-sectional study, the postmenopausal women (aged 50–60 years) with higher lycopene intake in their dietary records had higher serum lycopene (*p* < 0.02), which reduces oxidative stress and bone resorption markers and may be beneficial in reducing osteoporosis risk [92].

## 3. Chicken Eggshell as Source of Calcium and Its Application

**Figure 1:**
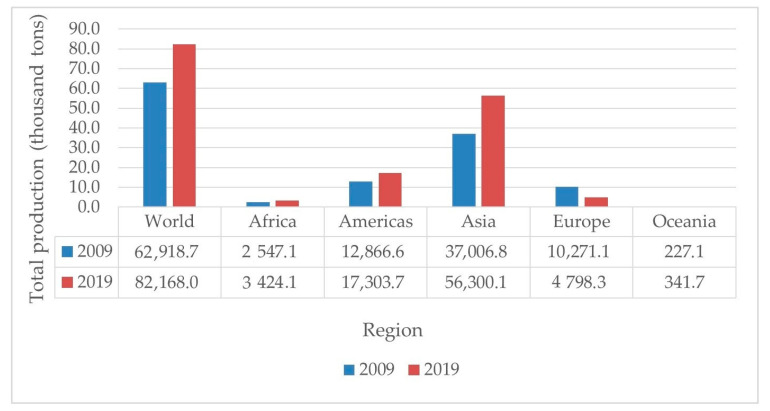
Total chicken egg production in the world and some regions (2009 and 2019) [93].

The increasing egg production in the world from 2009 to 2019 was observed (more than 30%) and is shown in Figure 1 [93]. In 2008, the egg production in European Union countries was in second place with more than 6.5 million tons, between China in the first place and the USA in the third-place [94]. In Poland, the average annual consumption of chicken eggs was more than 200 units per capita [95]. Not only the high consumption of eggs but also from hatcheries, home and food industries contributed to a high amount of the eggshells as waste [96]. Waheed et al. [97] informed that the weight of eggshells is about 9–12% of the total egg weight. These eggshells could be an environmental pollution threat if they are not used and only become a waste.

As an effort to utilize the high amount of chicken eggshell waste, many studies reported that chicken eggshells are the potential to be a calcium source [96,97,98,99]. Nutritional intake, including adequate calcium intake (ranged from 200–1200 mg daily, especially after menopause), has been known as an excellent approach for the maintenance of healthy bone status at all stages, starting from early infancy [6]. The eggshell contains 95% CaCO_3_ along with other valuable components, for example, strontium and boron, which act as the key role in osteoporosis prevention [96]. Around 37–39% of calcium can be isolated from chicken eggshell [100]. The composition of eggshell was reported in prior studies (Table 1).

Eggshell quality is affected by numerous factors, e.g., bird’s strain or age, induced molt, heat and general stress, diseases, production system, and addition of proprietary products to the diets [101]. Therefore, nutritional factors, such as feed composition (calcium, phosphorus, vitamins D, C, E and A, non-starch polysaccharides, enzymes and contaminants, e.g., mycotoxins) and water quality strongly influence the composition of both eggshell and internal egg quality. The results of many studies concerned the effect of ingredients of plant origin on the quality of eggs, where their influence on the composition of the inner part of the eggs was confirmed, while the composition of the eggshell was not analyzed, except for their physical parameters [102,103,104,105].

**Table 1:** Composition of chicken eggshells.

Specific Minerals Present in the Eggshell	Unit of Measurement	According to the Prior Research			
		[106]	[107]	[98]	[108]
Total ash content	g/100 g	89.9–91.1	90.2	N.D. ^1^	N.D.
Calcium	mg/100 g	35,100–35,400	35,080	38,200	40,100
Magnesium	mg/100 g	370–400	262.0	N.D.	450
Iron	mg/100 g	N.D.	13.06	N.D.	2.24
Phosphorus	mg/100 g	120	150.2	N.D.	99
Zinc	mg/100 g	N.D.	145.1	N.D.	0.513
Sodium	mg/100 g	150–170	47.9	510	N.D.
Potassium	mg/100 g	100–130	50.00	140	N.D.
Copper	mg/100 g	N.D.	4.1	N.D.	0.77
Manganese	mg/100 g	N.D.	149.9	N.D.	N.D.
Strontium	μg/g	N.D.	N.D.	140	372
Fluorine	μg/g	N.D.	N.D.	N.D.	3.75
Selenium	ng/g	N.D.	N.D.	N.D.	23.5

^1^ N.D. = not determined.

Chicken eggshell powder could be a good method to increase the calcium intakes across rural sub-Saharan Africa, where calcium intake is low, and over 85% of rural households in sub-Saharan Africa keep poultry [99]. The bioavailability of chicken eggshell calcium is also as good as CaCO_3_. A study with rats reported that the calcium absorption from a diet containing chicken eggshell powder was 45.59 ± 14.43%, not significantly different with supplement CaCO_3_ (39.88 ± 16.07%) (*p* > 0.05) [98]. The applications of chicken eggshell as the source of calcium in various food products are shown in Table 2. The use of eggshells can bring significant benefits in terms of nutritional value and, above all, an increase in calcium content. However, it should be noted that the success of adding eggshells depends on the type and pH of the food product. Its addition to bread and biscuits depends on the desired level of calcium, the acceptability of the organoleptic characteristics of the product, but above all, from the technological point of view, the increase of rheological characteristics of dough, the volume of the dough, and the texture of the final product. In sweet confectionery products, the addition of eggshells may adversely affect the sensory characteristics and acceptability both in terms of taste and aroma. The addition of eggshells to dairy products, such as yogurt, fried cheese or cranberry juice, seems to be the most beneficial and sensory desired. The application of eggshells in the food industry is also limited due to high microbiological contamination, e.g., with *Salmonella*, and the need to grind the shells appropriately so that the consumer does not perceive its presence as “sand” in the product.

**Table 2:** Application of chicken eggshell powder in food products.

Food Products	Main Findings		References
	Chicken Eggshell Powder Concentration	Notes for Recommendation	
Biscuits	6% (*w/w*) of wheat flour	Calcium content, texture, sensory properties, calcium bioavailability	[107]
Bread	8% (*w/w*) of ingredients	Calcium content, specific volume of bread, sensory properties	[109]
Bread	2% (*w/w*) of wheat flour	Increase of rheological characteristics of dough and nutritional properties, decrease of the general acceptability and odor score	[20]
Bread strips	10% (*w/w*) substitution of wheat flour	Minor changes in sensory properties	[18]
Breaded fried meat, bread, pizza, spaghetti	500 mg Ca/person	Minor changes in texture, without flavor changes	[98]
Chocolate cakes	6% (*w/w*) of wheat flour	Calcium content, texture, sensory properties	[110]
Chokeberry juice, cranberry juice	1% of chokeberry and cranberry juice	Calcium content of chokeberry and cranberry juice, no significant change in color and sediment content	[69]
Muffin	8 g/500 g wheat flour	Mineral content, sensory properties	[111]
*Nham* (Thai-style fermented pork sausage)	150 mg Ca/100 g of *Nham* (eggshell powder was converted to eggshell calcium lactate)	No difference in sensory scores of sour taste, flavor, and overall acceptance	[112]
*Ser smażony* (Polish bread spread)	265 mg/100 g of *ser smażony*	Increased calcium contents >2.5-fold, the calcium bioavailability was higher after the addition of lysine and vitamin K	[113]
White bread	1–1.5% (*w/w*) of ingredients	High total score in sensory evaluation	[114]
White bread	2% (*w/w*) substitution of bread flour	Consumer acceptation in sensory evaluation	[115]
Yogurt	0.15–0.30% (*w/v*) of milk	No significant unfavorable effects on the physicochemical, microbial, and sensory properties	[116]
Yogurt (from cow milk and buffalo milk)	0.3% of yogurt (nanosized eggshell powder)	Acceptable composition, texture, and sensory attributes	[117]

### Correlation between Tannic Acid and Calcium Absorption

The effect of tannin on the bioavailability of calcium was studied. According to recent research, tannin was reported to be an inhibitor of calcium bioavailability [83,118]; however, the results of Proulx et al. [119] did not support this and reported that tannins do not affect calcium bioavailability. According to Amalraj and Pius [118], increasing the concentration of tannic acid results in a higher decrease of calcium salts bioavailability. The highest percentage of decrease was found in calcium formate (28.1%), and the lowest in calcium gluconate (19.6%), with the ratio of 1:1 between calcium salt and tannic acid. Gupta et al. [120], who studied the effect of the bioavailability of calcium and iron from selected green leafy vegetables, did not confirm that only tannic acid alone decreases the bioavailability of calcium from green leafy vegetables. They found that the calcium bioavailability was influenced by the presence of different inhibitory factors (such as oxalic acid, phytic acid, polyphenols, and dietary fiber). However, Proulx et al. [119] reported that tannin content does not affect the calcium bioavailability of legumes. It has been found that tannins may inhibit iron bioavailability to a greater extent than the bioavailability of calcium.

The application of chicken eggshell powder in tannic acid-rich fruits, chokeberry and cranberry juice was once reported by Lachowicz et al. [69]. In their article, they did not mention the inhibition of calcium absorption by tannic acid from chokeberry and cranberry, but they were concerned that the low pH in chokeberry and cranberry juice could increase the solubility of calcium from chicken eggshell powder, then afterward could increase the calcium absorption. Furthermore, the same authors concluded that CaCO_3_ in natural form is much more soluble than synthetic CaCO_3_, and the porous structure of CaCO_3_ in chicken eggshell could also increase the calcium solubility.

## 4. Factors Supporting Calcium Absorption

Not only the high amount of calcium but also the calcium absorption is important. Some factors that influence calcium absorption positively were reported, including an acidic condition in the intestine (especially for CaCO_3_ absorption), estrogen, vitamin D, and soluble fiber/prebiotics (inulin, fructooligosaccharose, oligofructose), probiotics, and synbiotics [121,122,123]. Studies on the bioavailability of calcium from eggshells in humans are scarce, however, showing its potential. Schaafsma and Pakan [124] studied the short-term effects on the bone mineral density of the lumbar spine and hip in humans who consumed dairy-based supplements with eggshell powder. Results demonstrated that bone mineral density of the lumbar spine, total proximal femur and trochanter were significantly increased, showing that eggshell powder is a source of bioavailable calcium. Other research on the effect of eggshells consumption showed that healthy late postmenopausal women with proper calcium intake at baseline might increase the bone mineral density of the hip within a year of supplementation [125].

### 4.1. Vitamin D

There is a “calcium paradox”, which describes that hip fracture incidence is higher in western countries, where the diet is rich in calcium, while in developing countries, where calcium is poorly included in human diets, the occurrence of these fractures is lower. This paradox may be partially understood considering the content of vitamin D in the serum of the local population [126].

Vitamin D is necessary for calcium and bone metabolism, as it is the principal factor that maintains calcium homeostasis and absorption [126,127]. Deficiency of vitamin D can accelerate bone loss, increased bone turnover, osteoporosis and fractures [128]. Elderly people are at high risk of vitamin D deficiency due to limited sources of vitamin D in the diet, less sunlight exposure, decreased capacity to synthesize vitamin D in the skin and decreased capacity of the kidneys to convert vitamin D into the active form [129]. There are two major forms of vitamin D. First is vitamin D2 (ergocalciferol), which is derived from UV-B radiation of ergosterol, the vitamin D precursor naturally found in plants, fungi, and invertebrates. The second is vitamin D3 (cholecalciferol), which is originated by sunlight exposure from 7-dehydrocholesterol, a precursor of cholesterol that can also act as provitamin D3 [126]. Although major vitamin D (90%) is endogenic (synthesized in the skin from UV irradiation), vitamin D can also be found in some food sources, including eggs, meat, seafood (salmon, tuna, sardines, herring), mushrooms and dairy products [121]. It is essential to concern about the food sources for vitamin D because the rate of cutaneous vitamin D synthesis is reduced in elderly people, which puts them at risk for vitamin D deficiencies, which are related to bone fractures [130].

The activation of vitamin D is needed in the human body. Serum 25-hydroxyvitamin D (25OHD), which acts as intermediate compounds before forming the final biologically active vitamin D, is a marker allowing the estimation of a person’s vitamin D nutritional status [121,131,132]. The 25OHD amount less than 27–30 nmol/L is associated with rickets, while the level of 25OHD at least 50 nmol/L (20 ng/mL) is categorized as sufficient, even though the optimal level for intestinal calcium resorption is about 80 nmol/L [121,133]. Vitamin D undergoes hydroxylation in the liver to 25OHD and later in the kidney is converted into 1,25-dihydroxy vitamin D, which is the most active metabolite stimulating calcium and phosphate absorption from the gut, which also influences bone cells [126,134].

Some research about vitamin D, especially to enhance calcium absorption in the elderly were reported. Meta-analyses indicated that vitamin D supplementation alone is unlikely to reduce fracture risk; calcium supplementation alone has a moderate effect in reducing the fracture risk (around 10%), but in the long-term, the fulfillment of calcium supplementation is poor [135]. However, the combination of vitamin D and calcium supplementation, especially in people at risk of marginal and low vitamin D status (<60–80 nmol/L serum 25OHD), reduces the total fractures, including hip fractures [135]. A study reported that 1000 mg calcium citrate/day and vitamin D at a dose equivalent to 600 IU/day given over a year improved the bone mineral density or bone markers, not significantly different from the higher vitamin D dose of 3750 IU/day in overweight elderly with a mean 25OHD of 20 ng/mL [131]. Taylor [136] included vitamin D and calcium-enriched food as one of the functional food types for the elderly, such as fruit juice, soya milk, cheese, milk, and yogurt enriched with vitamin D and calcium. According to European Food Safety Association [137], 800 IU or 20 μg of daily vitamin D intake and 1200 mg of daily calcium intake are recommended for women aged 50 years and older to reduce bone loss and maintain bone mineral density. Nowson [135] reported that the elderly are recommended to consume >1100 mg/day of calcium together with maintaining adequate vitamin D status (>60 nmol/L 25 OHD) to reduce the risk of fracture.

### 4.2. Prebiotics, Probiotics, and Synbiotics

Prebiotics are nondigestible fibers, which acts as the substrates for microbes inside the gut to ferment the nutrients and energy sources and can also improve the viability of gut microbes [138]. Many studies reported about the role of prebiotics in increasing calcium absorption, thus may reduce the risk of osteoporosis. Prebiotics, such as fructans (with dosage 4 to 8 g/day), including inulin, oligofructose, and fructooligosaccharide, enhance calcium absorption [7,121]. These fructans are fermented by gut microbes, and the product of this fermentation decrease the pH in the colon, which can increase macromineral absorption [121], including calcium, as in the acidic environment, calcium is converted to ionic form and becomes more soluble and bioavailable [138,139]. The prebiotic fiber fermentation products, which produce an acidic environment, are the short-chain fatty acids, which, together with low pH, resulting in the hypertrophy of the mucosal cells, which leads to an enlargement of the surface area of the intestine and thus improve the absorption of calcium [139].

Prebiotics can stimulate the growth of gut microbes, which is beneficial for the elderly, with a significant decrease in the amount of bifidobacteria [7,139], thus can affect the bone health of the elderly. The application of prebiotics in food products high in calcium is the potential to reduce the risk of osteoporosis in the elderly. By using in vitro experiment, Krupa-Kozak et al. [140] analyzed that inulin-type fructans, especially short-chain fructooligosaccharide, significantly increased cellular (intestinal-like Caco-2 cells) calcium uptake from calcium-enriched gluten-free bread digest and stimulated the intestinal bacteria (*Lactobacillus*, *Enterococcus*, and Enterobacteriaceae) applied in the cultures to the intensive synthesis of organic acids (*p* < 0.05). Jakeman et al. [141] found that daily soluble corn fiber intake significantly increased bone calcium retention in postmenopausal women and improved the bone calcium balance by an estimated 50 mg/day. In rats studies, Arora and Patel [142] compared the calcium absorption of skimmed milk enriched with fiber Blend-I (psyllium husk, oat fiber, MCC, inulin), fiber Blend-II (psyllium husk, oat bran, wheat fiber and inulin), and control (cellulose). The calcium absorption of fiber Blend-I, fiber Blend-II, and cellulose was 94.4 ± 1.9%, 96.3 ± 1.32%, and 88.8 ± 4.9%, respectively. However, the difference in calcium absorption between groups was not significant (*p* > 0.05), indicating that these dietary fibers can be incorporated into dairy products to improve the nutritional value.

Research about the effect of probiotics on calcium absorption has been conducted. Some mechanisms by probiotics, which lead to increase the bioavailability of mineral, especially calcium, include (1) increasing the mineral solubility by producing the short-chain fatty acids and simultaneously decreases the parathyroid hormone (PTH) level (increasing PTH level causes the bone resorption by stimulating the osteoclast); (2) producing phytase by bacteria to overcome the effect of mineral depressed by phytate; (3) reducing intestinal inflammation, followed by increasing bone mass density; (4) hydrolyzing glycoside bond food in the intestines by *Lactobacillus* and *Bifidobacterium*; and (5) synthesizing vitamins, which influence the absorption of calcium [123,143]. Gilman and Cashman [144] reported that *Lactobacillus salivarius* significantly improved the calcium uptake into Caco-2 cell monolayers after 24 h (*p* < 0.05). Group of postmenopausal women taking the probiotic consisting of three strains: *Lactobacillus paracei* 8700:2, *Lactobacillus plantarum* Heal 9, and *Lactobacillus plantarum* Heal 19, showed a reduction in bone mineral density loss compared to placebo with 0.71% of average difference (*p* < 0.05) [145]. Although much research showed significant results, Scholz-Ahrens et al. [146] mentioned that probiotics alone did not significantly affect bone mineralization and gut ecology.

The combination of prebiotics and probiotics, also known as synbiotics, has been reported to work very well [147]. As mentioned before, the probiotic alone did not significantly affect bone mineralization and gut ecology [146]. The prebiotics-fed animals had significantly higher calcium absorption values, as rats on prebiotics had significantly higher amounts of cecal contents and lower pH in cecal and colonic contents, and their calcium balance tended to be increased (*p* < 0.01), but synbiotics were proven to give more synergistic effect on bone mineralization [144,148]. However, Klobukowski et al. [149] in rats study reported that no significant difference of calcium absorption was observed between white cheese with probiotics (*Lactobacillus plantarum*), with synbiotic (*Lactobacillus plantarum* and 2.5% inulin HPX), and control (without probiotic or prebiotic).

## 5. Eating Preference and Dietary Pattern of Elderly

In designing functional food products for the elderly, it is important to know their food preference and dietary pattern. In the elderly population, aging may affect different aspects of oral physiology and eating behavior. In terms of food oral processing, elderly people expect the ability to form and swallow the food bolus, while in terms of food sensory, they concern about the texture, taste and less pain sensation [150,151]. Difficulty swallowing the foods (dysphagia) affected 7% to 10% of people aged over 50 years [7]. Furthermore, some factors, such as the amount of money, household composition, psychological factor (fears associated with aging, including losing a mate, changing roles, moving, rejection, making new friends, and experiencing illness), society and culture/tradition, simple-cooking method, and sensory aspect of food affect the food preference of the elderly [152,153]. A study using a cross-sectional survey reported that trying to eat healthily is one of the highest main factors influencing the food choices of the European elderly, while the main benefits of healthy eating of the elderly are to stay healthy, to prevent disease, and to promote quality of life [154].

Regarding those factors, many studies reported about the dietary patterns of the elderly in many regions of the world. According to Briley [152], the most common foods consumed by the American elderly were white bread, ground coffee, whole milk, sugar, potatoes, tea, orange juice, eggs, butter, and bacon. A cross-sectional study based in the USA also reported about “Western-like” dietary pattern (high intake of bread, eggs, fried vegetables, and fats, and low intake of dairy products) in the elderly older than 75 years old [155]. In Brazil, South America, the “coffee with milk and bread and butter” pattern was associated with the elderly aged 80 years and older and elderly people with chewing problems [156]. Capurso and Capurso [157] suggested elderly, especially in Southern Europe, consume whole wheat sourdough bread as sourdough fermentation lowers the glycemic index of bread, and sourdough bread also provides vitamin E, vitamin B1, B6, B12, thiamin, niacin, folate, riboflavin, potassium, zinc iron, magnesium, selenium, calcium, phosphorus, and manganese. In terms of fruit preference between European elderly, Polish elderly had a higher percentage (38%), followed by Finnish elderly (26%), in eating fruits, including berries (fresh and processed form) if compared to other European countries [158]. Factors such as simple-cooking, tradition and sensory aspects influenced the Italian elderly to prefer eating traditional Italian dishes, including lasagna, pasta and bean soup, and risotto with saffron [153]. The vegetable and bread pattern was more common among grandparents living with children and those living in towns or urban areas in Botswana, Africa [159]. In South Korea, Asia, Shin et al. [160] studied that vegetables, fruits, kimchi (Korean traditional fermented cabbage), fish, tofu, rice cakes, dairy products/eggs, meat, noodles, seafood, poultry, and bread/cookies were highly preferred by the elderly in Seoul. Shin et al. [160] also reported that the elderly prefer “ready-to-eat” food (53.9%), “ready-to-cook” food (21.2%), “ready-to-heat” food (15.8%) and “fresh-cut” products (9.1%). Folic acid fortification in wheat flour for bread-making (2–3 mg of folic acid per kilogram of wheat flour, or about 0.13 mg of folic acid per 100 g of bread), which is required by Australian New Zealand Food Standards since 2009 [161], provides good impacts on Australian population [162]. As bread is one of the sources of dietary intakes of elderly Australians, this folic acid fortification was reported to be associated with significantly reduced plasma homocysteine levels in elderly Australians [163]. In summary, bread products are generally preferable by the elderly in the world from different regions. Additionally, bread products are suggested to be consumed in a high amount in the food pyramid and also have a low environmental impact [164].

## 6. Functional Foods for Elderly

Functional foods in the elderly diet can help to improve their quality of life, as well as to reduce the risk of diseases or delay the onset of serious diseases [165]. The functional foods to support the health properties of the elderly have been studied during the last decades. Generally, a dietary suggestion for the elderly is not much different from which for younger adults, but the aging-related physiological changes raise additional challenges [136]. These physiological changes can affect the ability to eat and digest the food, including a decrease in the secretion of saliva, stomach and pancreatic juices, insulin, and bile [7]. These problems may lead to deficiencies of nutrients in the elderly. Merely adding the serving sizes or meal frequency more is usually not working successfully in the elderly due to these physiological problems with eating and decreased appetite [166]. Therefore, many studies designed and recommended various types of functional foods that can help the elderly in improving their nutritional status and preventing deficiencies.

Taylor [136] and Jędrusek-Golińska et al. [7] recommended elderly to consume functional foods containing and/or enriched with dietary fiber; omega-3 polyunsaturated fatty acids; phytoestrogens; polyphenols; carotenoids (including α-carotene and β-carotene); lutein and zeaxanthin; prebiotics, probiotics, and synbiotics; plant sterols and stanols; calcium and vitamin D. According to Meydani [167], functional food enriched with vitamin E, an antioxidant nutrient, in an amount above recommendations may reduce the risk of cardiovascular disease, improving immune status, and modulate important degenerative condition related to aging. As the elderly people possess fewer lactic acid bacteria, especially bifidobacteria and higher populations of fungi and enterobacteria than younger adults, some studies suggested elderly consume probiotics to improve the microflora balance in the gut, reduce the risk of enteric infection, urinary tract infections, hypertension, and colon cancer [7,136,168]. Jędrusek-Golińska et al. [7] also reported that probiotics possess the ability to regulate the immune system, showed by a modest increase in prostaglandin E2; however, no changes were found in IgA levels in the elderly (*n* = 51, aged 65 years and older) using *Lactobacillus acidophilus*. Moreover, McCabe et al. [169] examined that *Lactobacillus reuteri* (ATCC PTA 6475) could affect the intestinal TNF-α levels and enhance the bone density of 14-week old male mice after 4 weeks of treatment. A study suggested protein-enriched functional foods for the elderly as increased protein intake has been associated with beneficial health effects for the elderly, including faster rehabilitation after hip fractures, increased lean body mass and strength, and lowered risk of becoming frail [166]. Taylor [136] recommended vitamin D and calcium-enriched functional foods to promote bone health and prevent osteoporosis in the elderly.

## 7. Conclusions

The effort to reduce the risk of osteoporosis in the elderly can be done through functional food high in antioxidant and calcium sources. The sources of antioxidants are various, including vitamin C, vitamin E, polyphenols, and lycopene. Intake of calcium is also important, and many researchers reported that chicken eggshell powder has a high content of calcium and can be consumed daily through its application in food products. The usage of eggshells as a source of calcium is also beneficial for the environment. Furthermore, the number of antioxidants applied in the food product must be concerned as well, so it would not interrupt the calcium absorption or could be a prooxidant, which negatively affects bone health. The addition of vitamin D, prebiotics, probiotics, and synbiotics could help to increase calcium absorption.

Considering the food preferences and dietary pattern of the elderly from around the world, the food waste utilization, and the reported application of chicken eggshell in various food products with their sensory analysis, it was suggested that the application of antioxidant and calcium from chicken eggshell powder is potential to reduce the risk of osteoporosis in the elderly.
